# A 3D-Printed Soft Fingertip Sensor for Providing Information about Normal and Shear Components of Interaction Forces

**DOI:** 10.3390/s21134271

**Published:** 2021-06-22

**Authors:** Gerjan Wolterink, Remco Sanders, Bert-Jan van Beijnum, Peter Veltink, Gijs Krijnen

**Affiliations:** 1 Robotics and Mechatronics Group (RAM), University of Twente, 7500 AE Enschede, The Netherlands; r.g.p.sanders@utwente.nl (R.S.); gijs.krijnen@utwente.nl (G.K.); 2 Biomedical Signals and Systems (BSS), University of Twente, 7500 AE Enschede, The Netherlands; b.j.f.vanbeijnum@utwente.nl (B.-J.v.B.); p.h.veltink@utwente.nl (P.V.)

**Keywords:** 3D-printing, conductive, TPU, flexible, soft, shear force, fingertip sensor

## Abstract

Sensing of the interaction forces at fingertips is of great value in assessment and rehabilitation therapy. Current force sensors are not compliant to the fingertip tissue and result in loss of touch sensation of the user. This work shows the development and characterization of a flexible fully-3D-printed piezoresistive shear and normal force sensor that uses the mechanical deformation of the finger tissue. Two prototypes of the sensing structure are evaluated using a finite element model and a measurement setup that applies normal and shear forces up to 10 N on a fingertip phantom placed inside the sensing structure, which is fixed to prevent slippage. Furthermore, the relation between strain (rate) and resistance of the conductive TPU, used for the strain gauges, is characterized. The applied normal and shear force components of the 3D-printed sensing structure can be partly separated. FEM analysis showed that the output of the sensor is largely related to the sensor geometry and location of the strain gauges. Furthermore, the conductive TPU that was used has a negative gauge factor for the strain range used in this study and might cause non-linear behaviors in the sensor output.

## 1. Introduction

Measurement of the interaction forces between the fingertips and the external environment is of great value in upper extremity assessment and for interaction in rehabilitation therapy where patients need to relearn motor tasks [1]. While handling objects, these interaction forces can reach up to 50 N for normal and up to 10 N for shear forces, where the high dynamic range for resolving both forces is below 10 N [2,3]. Current available force sensors, capable of measuring shear and/or normal forces, show two main difficulties. Most sensors are mainly made of stiff materials leading to the loss of touch sensation of the user. Secondly, these sensors are not ideally adapted to the shape and stiffness of the fingertip, leading to poor sensor-to-skin attachment. Due to this poor contact the sensors tend to shift easily by external forces [4]. In practice this issue has presented itself in the form of limited usefulness and reliability of current force sensors [5].

In recent years the interest in and development of 3D-printed sensors have increased due to the advent of multi-material printers and increasing availability of conductive thermoplastic materials [6,7,8]. The introduction of soft and flexible materials with electrical properties creates the opportunity for applications in soft robotics [9]. Furthermore, the high customizability of 3D-printing makes these sensors an ultimate candidate for biomedical applications such as biopotential electrodes [10,11], tactile and bending sensors [12,13], and soft finger shear- and normal-force sensors [14]. Ideally, the sensor on the fingers should be soft and not affect the touch sensation of the user. These 3D-printed sensor structures have potential advantages for development of complex sensing structures that cannot be produced using traditional production methods, while still being highly customizable to fit to an individual user. Furthermore, these sensing structures may in the future be integrated into larger, multi-functional structures [7].

The goal of this work is to create and characterize a 2 degrees of freedom (DoF) force sensor, which measures the normal and interaction shear forces between the environment and the fingertips. To minimize the loss of touch sensation of the user, the sensor structure should be compliant with the soft finger tissue and ensure direct interaction between fingertip skin and the object.

## 2. Design

**Figure 1:**
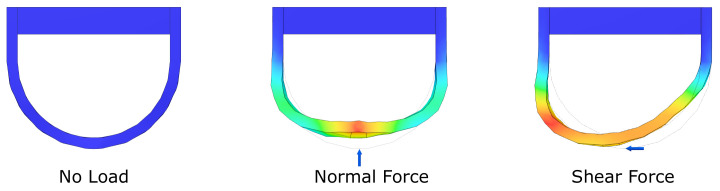
Deformation of the sensing structure under normal and shear force, colors represent amount of deformation.

The sensing principle of the sensor is based on the mechanical deformation of soft tissue around the bone (distal phalanx) in the fingertip [14]. A normal or shear force applied by the fingertips results in a movement and compression of the soft tissue relative to the distal phalanx and nail-bed. This movement can be sensorized by placing a flexible strap, with embedded strain gauges, on top of the nail and around the fingertip. Since a normal force exerted on the fingertip results in a symmetric deformation on both sides of the straps and a shear force induces a non-symmetric deformation (see Figure 1), the strap contains two strain gauges, one on each side, to separate normal from shear force.

Yin et al. [3] demonstrated a comparable setup using traditional fabrication methods using microfluidic channels filled with liquid metal. Their design focuses on robotic tactile sensing and therefore does not focus on facilitating the touch sensation of human fingertips, and the design and characterization lack the presence of the distal phalanx and nail-bed. Nevertheless, the work by Yin et al. supports the interest in these types of sensors in (soft) robotic tactile sensing or use in prosthesis for haptic feedback applications.

**Figure 2:**
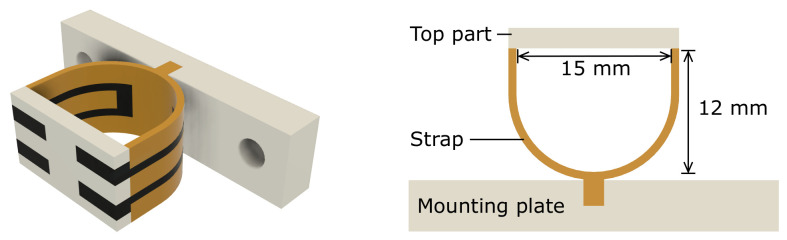
Render of the sensor design. Orange represents non-conductive TPU material, black represents the strain gauges made of conductive TPU material, and the rigid PETG parts are represented in gray.

The design and dimensions of the sensors are shown in Figure 2. The flexible strain sensors are made from carbon-doped conductive TPU (PI-ETPU, Palmiga Innovation, Jonstorp, Sweden) and are represented by the black-colored material. The orange flexible strap is made from non-conductive TPU material (NinjaFlex, Fenner Drives, Manheim, PA, USA) and has a width of 0.8 mm, being two traxels (track-elements).

To study the influence of the strain gauge location, two versions of the sensor were designed. One structure has the strain gauges placed on the inside (inside sensor), having only non-conductive material on the outside, while the second sensor has the opposite design with the strain gauges implemented on the outside (outside sensor).

The rigid top part and bottom mounting plate of the sensor are made from PETG (3DJake, Niceshops GmbH, Paldau, Austria) and are represented in gray. The bottom mounting plate can be mounted onto the test setup and is fixed to the center-line of the flexible strap of the finger sensor, allowing for shear force measurements without slip.

## 3. Numerical Simulation

**Figure 3:**
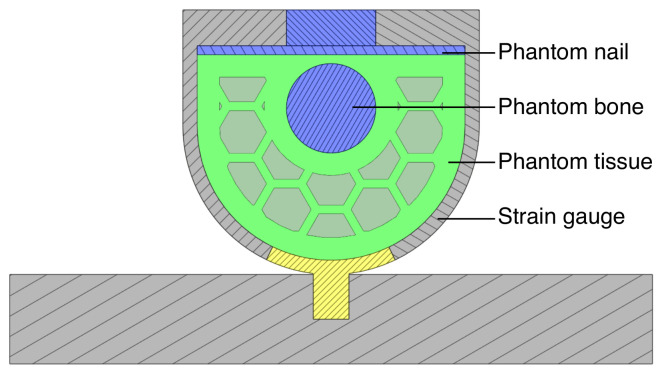
Cross-sectional view of finger sensor with phantom, printed with 30% honeycomb infill.

A finite element method (FEM) simulation was performed using a Static Structural Analysis in Ansys Workbench (ANSYS Inc., Canonsburg, PA, USA) to estimate the strain induced by normal and shear forces on the inside and outside of the sensor’s geometry. A cross-section of the finger model including the sensor is shown in Figure 3. To simulate the relatively low Poisson’s ratio of the soft finger tissue [15], the soft tissue part of the finger phantom is made from TPU with a 30% honeycomb infill. Both the conductive and non-conductive TPU materials were modeled using the five-parameter Mooney–Rivlin hyper-elastic material model that was fitted on experimental stress-strain data of the NinjaFlex TPU performed by Twank et al. [16]. The other materials were selected from the Ansys Engineering data library. The mechanical properties of the “finger bone” were modeled as stainless steel (316, annealed) due to the high stiffness of bone (17 GPa [15]) and to the chosen stainless steel fixture in the test setup; other stiff parts were modeled as PET (amorphous). Both the situation where only a normal force and the situation where only a shear force is applied were modeled by applying a 10 N sinusoidal remote force on the finger bone with a frequency of 0.5 Hz.

To estimate the strain and corresponding resistance change, a path along the curve of the strain gauge is taken. This path is subsequently divided into elements for which the directional changes in the *x*- and *y*-directions (dx and dy) are computed. Since there are two versions of the sensor, one path is taken along the inside surface of the strap and the other along the outside surface of the strap. These directional changes per element in the *x*- and *y*-directions are exported to Matlab (Mathworks Inc., Natick MA, USA) to calculate an estimation of the strain of the *i*th element ($\varepsilon_{i}$):

(1) $$\varepsilon_{i}=\frac{1}{L_{i0}}\sqrt{{\left(dx_{i+1}-dx_{i}\right)}^{2}+{\left(dy_{i+1}-dy_{i}\right)}^{2}}$$

where $L_{i0}$ is the initial length of element *i*.

**Figure 4:**
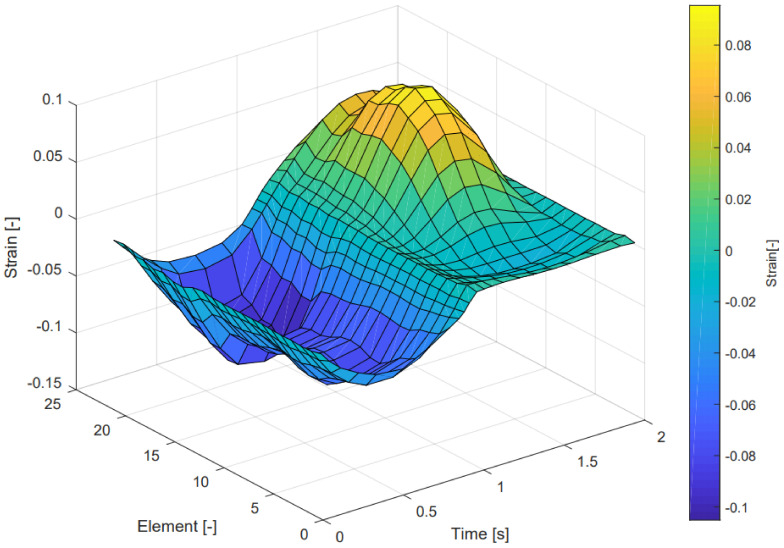
Estimated elemental strain as a function of time, per element of the outside path (only the path of one side of the strain gauge loop is taken). A 0.5 Hz sinusoidal shear force of 10 N was applied in the simulations.

The results of Equation (1) in the case of the outside path are visualized in Figure 4, showing the strain per element as a function of time.

Next, the resistance change of the whole path (ΔR/R) can be computed by multiplying the elemental strain with the gauge factor (GF) and initial resistance at zero load per element ($R_{0i}$):

(2) $$\frac{\Delta R}{R}=\frac{1}{R_{0}}\sum_{i=1}^{n-1}\varepsilon_{i}GFR_{0i}$$

The sum of the elemental resistance of the path ($R_{0}$) is set to 2.2 kΩ and a gauge factor (GF) to −15 for ETPU. Because the U-shaped strain gauges are symmetrical, only the path of one side of the loop is taken. For simplicity, it is assumed the connection pads and bottom loop only contribute as fixed mutual resistance.

## 4. Methods

Both the inside and outside sensor designs were evaluated using a linear actuator that applies forces to a 3D-printed phantom fingertip with the same design and materials as discussed in Section 2. Furthermore, the relation between the resistance and the strain rate of the conductive TPU were studied.

### 4.1. Characterization of Conductive TPU

**Figure 5:**
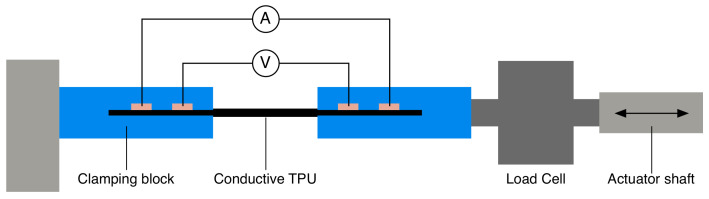
Setup to measure the resistance while straining a sample of conductive TPU. A constant current was applied on the sample while it was stretched at a constant rate using a linear actuator.

To investigate the influence of strain on the resistance of the conductive TPU filament used in the sensor model, a sample conductive TPU filament (ETPU 85-700+, Palmiga Innovation, Jonstorp, Sweden) with a diameter of 1.75 mm was loaded into a strain setup. In this setup the filament was clamped between two blocks containing copper strips to connect the sample in four point configurations to determine the resistance as shown in Figure 5. The distance between the two clamping points was 10 mm.

A current of 100 μA was supplied by a source measure unit (Model 2410, Keithley Instruments, Inc., Cleveland, OH, USA) and the voltage drop was recorded using a USB oscilloscope (Handyscope HS5, TiePie engineering, Sneek, The Netherlands). One side of the clamping blocks was fixed while the other side was pulled along the length direction of the sample using a linear actuator (SMAC LCA25-050-15F, SMAC, Carlsbad, CA, USA) position controlled by a computer using Matlab (Mathworks Inc., Natick, MA, USA). The force was measured by a load cell (LCMFD-50N, Omega Engineering, Norwalk, CT, USA) placed between the actuator and the clamping block. The output of the load cell was amplified (IAA100, Futek, Irvine, CA, USA) and connected to the USB oscilloscope together with the encoder output of the linear actuator to synchronize with the resistance change.

The samples were strained at a strain rate of 1 ε/min until a length of about 140%, after which the sample was unloaded at the same rate. After 10 cycles the sample of conductive TPU filament was replaced with a fresh sample and the experiment was repeated at strain rates of 5, 10, and 20 ε/min.

### 4.2. Sensor Fabrication

**Figure 6:**
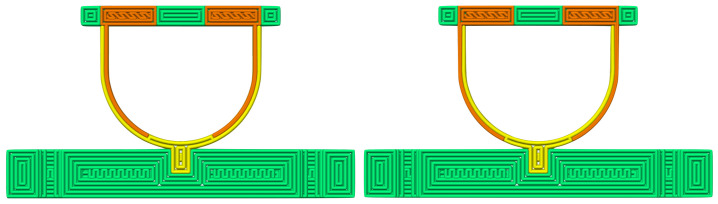
Top view of printing pattern of the inside (**left**) and outside (**right**) sensors. The conductive TPU is shown in orange, non-conductive TPU in yellow, and PETG in green.

**Figure 7:**
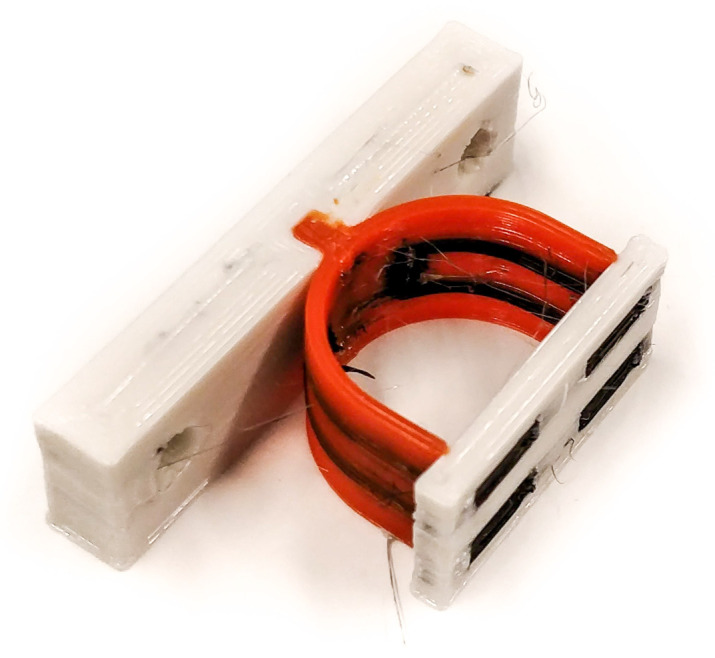
Photograph of the printed sensor with the strain gauges positioned on the inside. The conductive TPU parts are shown in black, non-conductive TPU in orange, and the PETG parts in white.

The strap of the sensors has a thickness of two traxels. Two versions of the sensor were printed, the first having the strain gauges positioned on the inside traxels of the strap (inside sensor); the second has the strain gauges positioned on the outside (outside sensor) as shown in Figure 6. The sensor was printed in one go using a Diabase H-Series Hybrid (Diabase Engineering, Longmont, CO, USA) multi-material FDM 3D-Printer. Figure 7 shows a photograph of the printed inside sensor. Control of the printer and printer settings was handled by the slicer software (Simplify 3D, Inc., Cincinnati, OH, USA). The layer height of the structure was set to 200 μm. After printing, the sensor was annealed for 15 h at 80 °C. Electrical interfacing to the conductive TPU of the sensor was made by melting a fine stranded copper wire into the connection pads of the sensor. The initial resistance of the strain gauges measured at this interface was in the range of 5 kΩ.

### 4.3. Sensor Characterisation

The finger phantom was built from a low infill (30%) honeycomb structure made of TPU, representing the soft tissue, printed in one go, together with a 1 mm-thick PETG part, representing the nail. The finger bone is represented by a stainless steel bolt with a diameter of 5 mm.

**Figure 8:**
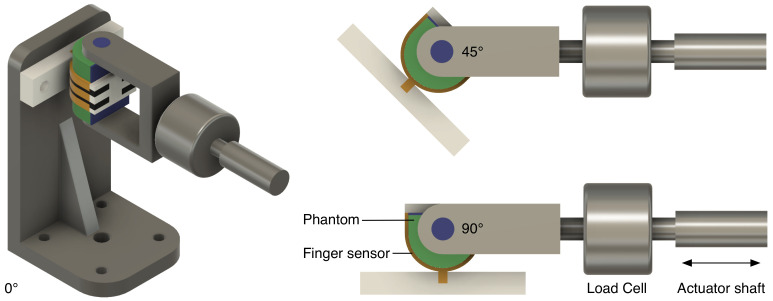
Measurement setup: the finger sensor and phantom can rotate around the phantom bone allowing the linear actuator to apply both normal and shear force components.

The force was applied to the stainless steel bolt by a mounting bracket connected to the shaft of the linear actuator (SMAC LCA25-050-15F, SMAC, Carlsbad, CA, USA). To apply both normal and shear force components to the phantom the actuator can rotate, with its center of rotation around the bold through the phantom, from −90° to 90°, where only a shear force is applied and at 0° only a normal force is applied. The force applied by the linear actuator was measured by a load-cell in line with the actuator shaft (LCMFD-50N, Omega Engineering, Norwalk, CT, USA). The measurement setup is shown in Figure 8.

The resistance change of the strain gauges was read out using a Wheatstone bridge configuration. Each strain gauge was connected separately in a quarter bridge configuration, the applied bridge voltage was 5 V and the output of the bridge was measured using an instrumentation amplifier (AD620, Analog Devices, Norwood, MA, USA). The amplified signal, representing the resistance change of each strain gauge, was logged together with the encoder output of the linear actuator and the output of the load-cell amplifier (IAA100, Futek, Irvine, CA, USA) using a digital oscilloscope (Handyscope HS5, TiePie engineering, Sneek, The Netherlands).

The sensor was actuated under multiple angles to apply both shear and normal force components. At 0° the only force component is the normal force and at ±90° there is only a shear force component. Under each angle the actuator produces a sinusoidal force of 0.5 Hz with an amplitude of 10 N for 20 s. The oscilloscope data were sampled at 200 kHz to capture all the encoder pulses and downsampled by a factor 10 for further analysis. Next the data from the load-cell and finger sensor strain gauges were low-pass-filtered using a 40 Hz second-order Butterworth filter to remove any high-frequency noise from the data. The finger sensor data were also passed through a 0.5 Hz second-order Butterworth high-pass filter to remove the drift. Next the normalized sensor output was obtained (ΔS) by subtracting the mean of the absolute value of the whole dataset of that sensor ($\Delta R_{\mathrm{mean}}$) from the measured resistance change ($\Delta R_{\mathrm{meas}}$) and subsequently dividing the result by $\Delta R_{\mathrm{mean}}$ (see Equation (3)).

(3) $$\Delta S=\frac{\Delta R_{\mathrm{meas}}-\Delta R_{\mathrm{mean}}}{\Delta R_{\mathrm{mean}}}$$

Next the loading cycles were segmented and the mean and standard deviation from the force, position, and sensor output (ΔS) of these cycles were taken. To exclude potential unwanted discrepancies at the start and stop of the measurements, the first and last cycles were excluded from the average data.

## 5. Results

### 5.1. Characterization of Conductive TPU

**Figure 9:**
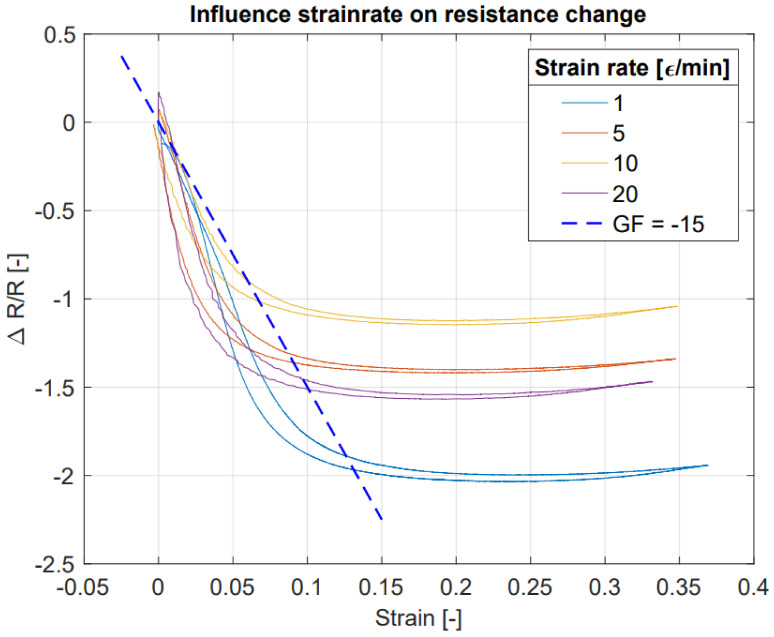
Average relative resistance change (ΔR/R) at various strain rates for ETPU filament: ΔR is given by the difference between initial resistance ($R_{0}$) and the measured resistance (*R*). The blue dashed line indicates the resistance change with a constant gauge factor (GF) of −15.

Figure 9 shows the average relative resistance change over eight cycles (second till second-last cycle) for several strain rates. The figure shows for each strain rate a decline in resistances up to a strain of around 0.2, after this point the resistance slowly increases with increasing strain.

**Figure 10:**
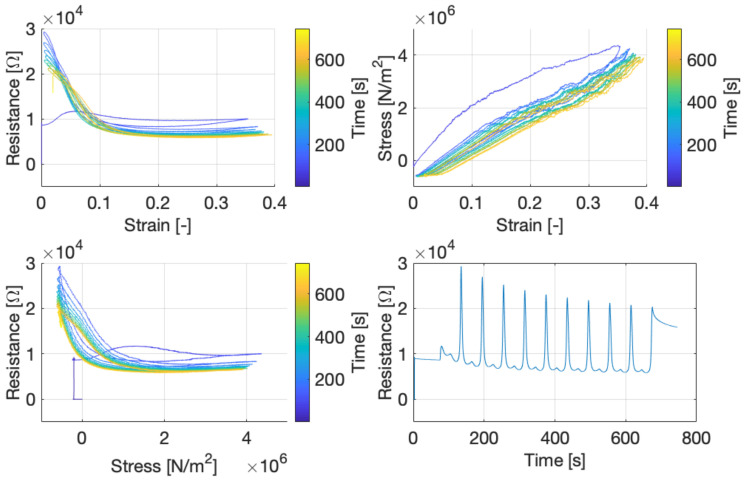
**Top left**: Resistance as a function of strain and time. **Top right**: Stress (initial diameter)–strain curve. **Bottom left**: Resistance as a function of stress and time. **Bottom right**: Resistance change over time during and 70 s after the measurement. These figures show all 10 strain cycles at a strain rate of 1 *ε*/min.

Figure 10 shows the recording of one sample that is strained ten times at a strain rate of 1 ε/min. The graphs clearly show the difference in the first cycle compared to the subsequent cycles. Furthermore, Figure 10 shows, in the bottom-right plot, that after the cyclic straining has been stopped and the material is back to the original position, the resistance is still clearly decreasing.

### 5.2. Force Sensor

**Figure 11:**
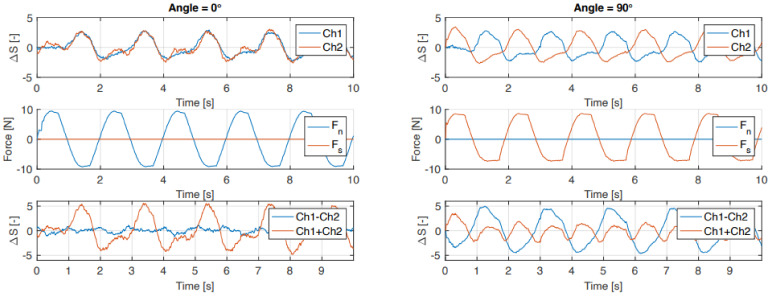
Time domain data of outside sensor for the first five force cycles where only a normal force (0°) and shear force (90°) are applied.

**Figure 12:**
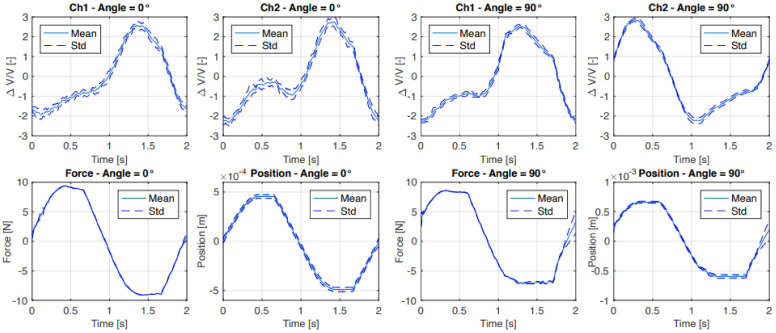
Averaged time domain data and standard deviation of the second until the ninth cycle where only a normal force (0°) and shear force (90°) are applied to the outside sensor. The position represents the position of the actuator shaft.

Figure 11 shows the time domain data for the outside sensors of the first five force cycles in the two extreme positions (0° and 90°). At 0° angle it is clearly visible that both strain gauges have very similar signal outputs, whereas at an angle of 90° the outputs of the two strain gauges are shifted by half a period. The standard deviation in Figure 12 shows that these cycles are repeatable. Furthermore, at 0°, it shows that the sensor is more sensitive to tension than to compression.

**Figure 13:**
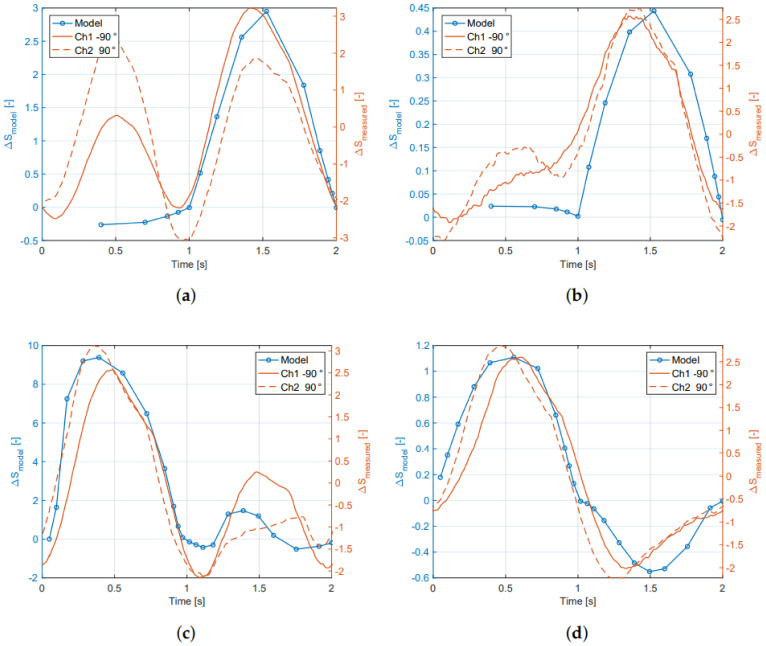
Average sensor response and the modeled (FEM) response of both the inside and outside sensors. (**a**) Normal force inside sensor; (**b**) normal force outside sensor; (**c**) shear force inside sensor; (**d**) shear force outside sensor.

The combined results for the average sensor response and the modeled response of both the inside and outside sensors are shown in Figure 13. For the normal forces Figure 13a,b show that the slope of the estimated resistance change from the FEM model is close to the recorded sensor output, mainly in situations where the strain-gauge is under tension. For shear force loading, Figure 13c,d, the shape and slope of the model, come close to the measured data in both negative and positive shear, especially for the outside sensor.

**Figure 14:**
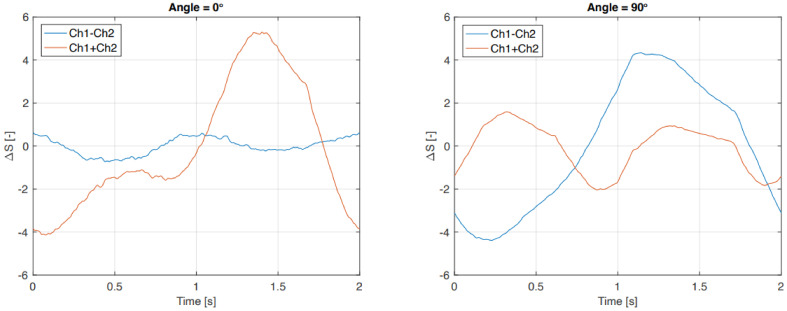
Sum and difference of the two strain gauges of the outside sensor in normal and shear situations.

**Figure 15:**
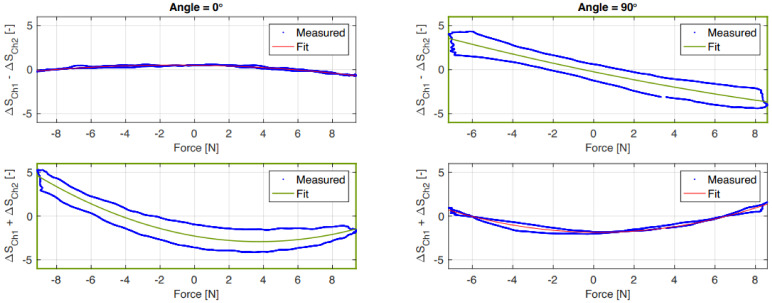
Relation of the outside sensor output sum and difference against normal force (0°) and shear force (90°). The fitted data indicated in green were used to estimate the normal (Fn) and shear force (Fs) components.

Figure 14 shows the sum and difference of sensors one and two of the averaged data for the outside sensor. The Figure shows that the difference (ch1–ch2) between the two sensors is not very sensitive to a normal force but clearly shows a stronger dependence on shear force. The relation of the sensor output sum and difference against normal force (0°) and shear force (90°) are shown in Figure 15.

**Figure 16:**
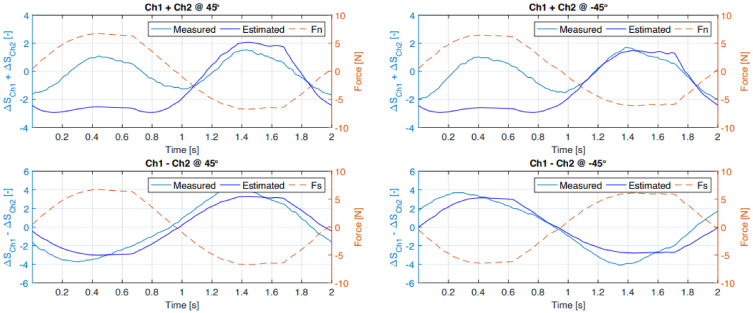
Estimated sensor output using the fit indicated in Figure 15 and the measured sensor output at 45° and −45° of the outside sensor.

The fitted data indicated in green in Figure 15 were used to estimate the sensor output in the 45° and −45° situation where the normal and shear force components are equal. These estimated data against the measured sensor output are shown in Figure 16. As indicated before, these data follow the applied shear force but in the normal force situation are limited to primarily sensing the tension force.

## 6. Discussion

### 6.1. Characterization of Conductive TPU

The results in Figure 10 clearly show that the first strain cycle deviates from the following cycles. This effect, shown in the stress–strain curve, is known as softening behavior [17], where in this case most softening happens in the first cycle. Furthermore, the conductive TPU materials seem to have a settling time when going back to the initial resistance at zero strain. This settling time is visible in the bottom-right graph of Figure 10 after 675 s, where after the last cycle the resistance of the material slowly decreases. This behavior is not uncommon for conductive polymer composite materials [8,18] and corresponds to the time-dependence stress–strain curves for TPU materials [17]. We note that the initial resistance drop at low strain values may be related to alignment of conductive carbon black particles in the TPU material, whereas at higher strains breaking down of these networks may be causing the increase in resistance, as proposed in [8]. Additionally, Rusinek et al. hypothesize the formation of new conductive pathways, caused by the contraction of the sample transverse to the strain due to Poisson’s ratio [19]. Furthermore, it is believed that the mobility of polymer chains causes the formation and breakdown of conductive networks [20], where at higher strain rates the resistance will increase more by this effect compared to lower strain rates since the breakdown effect is more dominant at high strain rates [20,21]. Although Figure 9 does not directly show this trend, there is a relatively large difference between 1 ε/min and the faster strain rates.

Christ et al. [8] have found the piezoresistive gauge factors to be similar for bulk material and FDM 3D-Printed samples. However, care has to be taken in the sensor design since the bulk material resistivity does not directly relate to the resistivity of a printed volume. Due to the nature of the FDM 3D-printing process the structures are built of track elements (traxels) that are stacked in layers. The contact interfaces between these traxels and layers result in anisotropic structures with the largest resistance in the direction of the stacked layers (*z*-direction) [22,23]. These effects should be taken into consideration while designing the printing pattern of conductive structures.

### 6.2. Force Sensor

Under loading of the normal force the strain gauges are bent at the edge and are not ideally compressed, making them less sensitive for compressive loading compared to tension forces where the sensors are strained. This is also expected on the basis of the FEM simulations. However, the actual sensors show higher sensitivity, especially in the inside configuration, than the simulated ones. The estimated output shown in Figure 16 also shows the lack of proper positive normal force estimation. Furthermore, the “parabolic” shape of the normal force ($F_{n}$) as a function of the sum of the sensor output shown in Figure 15 limits the possibility in an inverse model to distinguish between compression and tension forces. In a practical situation, where the sensor is placed on the fingertips to capture interaction forces, this might not be an issue since there are no tension force in the normal direction of the fingertips.

The output of the left and right sides of the strain gauges (Ch1 and Ch2 in Figure 11) should in theory be exactly similar due to the symmetry in the sensor design and measurement setup. However, experimental characterization shows some discrepancies between the two sensors. Besides possible inaccuracies in the measurement setup, we believe that the main cause is in the fabrication of the sensor due to inaccuracies in material extrusion during the printing process, material inhomogeneities, fluctuations in filament diameter, and variations in the bonding between traxels and layers.

The estimated resistance change by the FEM model is currently based on a constant negative gauge factor. However, the gauge factor of the ETPU material varies with the applied strain and strain rate as indicated by Figure 9. The FEM models of the strain gauge structures show the minimal and maximal strain per element to be −0.64 and 2.11 at the inside and −0.07 and 0.09 at the outside for the normal force situation. In the shear force situations the minimal and maximal observed strain per element were −0.86 and 2.88 for the inside and −0.11 and 0.10 for the outside sensor. However, these numbers are extreme situations for a single element. Furthermore, the inside sensor has higher values compared to the outside sensor in this FEM analysis due to friction between the strap and the phantom finger. The minimum and maximum observed strain of the complete paths of the strain gauge are −0.07 and 0.63 over all situations. As also visualized in Figure 4, a considerable contribution of the resistance change is caused by a negative strain (i.e., compression). The presented model assumes this compression will contribute to an increase in the strain gauge resistance as indicated in Figure 9. Although the comparison of the model outcomes to the experimental characterization show large similarities, this effect is not proven in a systematic experiment, dedicated to the behavior of the conductive TPU material under compression. Furthermore, in the performed strain test only an axial tensile strain was applied whereas in the sensor structure both compressive and tensile strain are present in multiple directions making the situation even more complex.

Another approach would be to measure the bending of the strap by measuring the difference between the strain on the inside and on the outside of the strap. This approach has been demonstrated by Tognetti et al. [24] using piezoresistive fabric to measure joint angles. These structures are not sensitive to elongation [24] and for example allow the finger sensor to be fitted pre-strained around the fingertip. Additionally, differential measurements have been shown to decrease non-linearities in the sensor response of 3D-printed piezoresistive sensors [25]. However, this differential strain gauge approach results in an at least 50% thicker strap due to the additional strain gauges and insulation, reducing the flexibility and thus reducing the touch sensation of the user.

In the FEM model the extreme inside and outside edges were selected, whereas the actual inside or outside sensor covers the complete area ranging from outer edge to the center line. Therefore, the physical sensors experience a slightly different, less extreme, strain profile compared to the results of the FEM model. Nevertheless, the FEM model, especially in the shear force cases, clearly shows that the curved shape of the sensing structure strain gauges contributes to the relation between sensor output and applied (shear) force.

The applied force in this experimental setup was limited to 10 N interaction forces while handling objects could reach up to 10 N for shear forces and up to 50 N for normal forces [26]. The high sensitivity range in the human touch sensation for both normal and shear forces goes up to 10 N [2,3]. Due to the non-linear effects on the resistance of the TPU material, the accuracy of the sensor is limited. Current research on compensation methods will help to improve the accuracy of these strain-sensitive TPU sensors [7]. These methods are, for example, based on analogous electrical models fitted to experimental data. In the future these sensor systems might be paired with smart algorithms or neural networks to exploit the full potential of the measured data. Nevertheless, without the use of these compensation methods and the limited sensitivity for positive normal forces, printed force-sensing structures already have the potential to be used in practical applications. Lifting an object like, e.g., a cup, will induce a shear force at the fingertips in the direction of the gravitational force of the object. This signal can be used for grasp detection in movement sensing systems [5]. Furthermore, the high level of customizability of 3D-printing allows for improved sensor geometries that, for example, will minimally cover the skin of the fingertip, allowing for direct skin contact with the object. Both the customizability and the compliance of the soft TPU materials that allow the tactile experience contribute to the high potential of 3D-printed wearable force sensors in a growing market for wearable flexible sensors [27].

## 7. Conclusions

This work demonstrates a working concept of a 3D-printed flexible sensor capable of measuring shear forces. The sensitivity of the current design for normal forces is limited when used as a finger sensor. The normal and shear force components can be separated. However, creating an inverse model is limited since this relation is non-invertible. A finite element model of the sensing structure and sensing setup shows that the output of the sensor is largely related to the chosen sensor design and strain gauge location. Further deviations probably originate from hysteresis and other non-linearities of the carbon-infused thermoplastic polyurethane that was used. Characterization of this material shows that the gauge factor of the material depends on the amount of strain and shows strong evidence of being time- and strain-rate-dependent. Within the range of strain estimated by the FEM model, the conductive material shows a negative gauge factor.

Future improvements of the sensor are to be made by using advanced algorithms for signal analysis, or neural networks, to use the full potential of the sensor output. 3D-printing offers low-cost and easy customization of sensing structures, which in combination with the soft materials makes these sensors a promising candidate for soft robotics and biomedical applications such as finger interaction sensing.
